# Long Non-Coding RNA Analysis: Severe Pathogenicity in Chicken Embryonic Visceral Tissues Infected with Highly Virulent Newcastle Disease Virus—A Comparison to the Avirulent Vaccine Virus

**DOI:** 10.3390/microorganisms12050971

**Published:** 2024-05-11

**Authors:** Yuxin Sha, Xinxin Liu, Weiwen Yan, Mengjun Wang, Hongjin Li, Shanshan Jiang, Sijie Wang, Yongning Ren, Kexin Zhang, Renfu Yin

**Affiliations:** 1 State Key Laboratory for Diagnosis and Treatment of Severe Zoonotic Infectious Diseases, Key Laboratory of Zoonosis Research, Ministry of Education, Department of Preventive Veterinary Medicine, College of Veterinary Medicine, Jilin University, Changchun 130062, China; shayx21@mails.jlu.edu.cn (Y.S.); liuxinx@jlu.edu.cn (X.L.); yanww22@mails.jlu.edu.cn (W.Y.); wangmj20@mails.jlu.edu.cn (M.W.); hongjin23@mails.jlu.edu.cn (H.L.); jiangss21@mails.jlu.edu.cn (S.J.); sijie21@mails.jlu.edu.cn (S.W.); renyn21@mails.jlu.edu.cn (Y.R.); zhangkx8221@mails.jlu.edu.cn (K.Z.); 2 College of Food Science and Engineering, Jilin University, Changchun 130062, China

**Keywords:** Newcastle disease virus, long non-coding RNA, virulence, immune, metabolism, growth and development, nervous system

## Abstract

There are significant variations in pathogenicity among different virulent strains of the Newcastle disease virus (NDV). Virulent NDV typically induces severe pathological changes and high mortality rates in infected birds, while avirulent NDV usually results in asymptomatic infection. Currently, the understanding of the specific mechanisms underlying the differences in host pathological responses and symptoms caused by various virulent NDV strains remains limited. Long non-coding RNA (lncRNA) can participate in a range of biological processes and plays a crucial role in viral infection and replication. Therefore, this study employed RNA-Seq to investigate the transcriptional profiles of chicken embryos’ visceral tissues (CEVTs) infected with either the virulent NA-1 strain or avirulent LaSota strain at 24 hpi and 36 hpi. Using bioinformatic methods, we obtained a total of 2532 lncRNAs, of which there were 52 and 85 differentially expressed lncRNAs at 24 hpi and 36 hpi, respectively. LncRNA analysis revealed that the severe pathological changes and symptoms induced by virulent NDV infection may be partially attributed to related target genes, regulated by differentially expressed lncRNAs such as MSTRG.1545.5, MSTRG.14601.6, MSTRG.7150.1, and MSTRG.4481.1. Taken together, these findings suggest that virulent NDV infection exploits the host’s metabolic resources and exerts an influence on the host’s metabolic processes, accompanied by excessive activation of the immune response. This impacts the growth and development of each system of CEVTs, breaches the blood–brain barrier, inflicts severe damage on the nervous system, and induces significant lesions. These observations may be attributed to variations in pathology. Consequently, novel insights were obtained into the intricate regulatory mechanisms governing NDV and host interactions. This will aid in unraveling the molecular mechanisms underlying both virulent and avirulent forms of NDV infection.

## 1. Introduction

Newcastle disease (ND), caused by virulent strains of avian paramyxovirus 1 (APMV-1, also named as avian orthoavulavirus 1, AOAV-1) and commonly known as Newcastle disease virus (NDV), is a highly contagious and often economically important disease found throughout the world that affects many domestic and wild avian species [1,2]. Based on the intracerebral pathogenicity index (ICPI) observed in day-old chicks, NDV can be classified into three pathotypes, known as lentogenic, mesogenic, and velogenic. Within these categories, velogenic NDV further differentiates into visceral or neurotropic types based on its tissue tropism, eliciting characteristic symptoms in infected poultry including hyperthermia, respiratory distress, neurological manifestations, and hemorrhagic lesions within the gastrointestinal tract. The mortality rate among susceptible birds can reach up to 100% [2]. On the other hand, lentogenic NDV infections only result in atypical symptoms like mild respiratory problems, along with slow growth and reduced egg production, and are generally followed by quick recovery [3]. Consequently, avirulent strains of NDV such as the LaSota strain are extensively employed as vaccines for prevention and control purposes against ND in clinical settings [4]. However, research regarding how both virulent and avirulent forms of NDV infection induce pathological damage within hosts remains limited.

Long non-coding RNA (lncRNA) is a class of RNA molecules that do not primarily encode proteins and have a transcript length exceeding 200 nt [5]. With the advances made in high-throughput sequencing technology, further lncRNAs have been continuously discovered, and an increasing number of studies have demonstrated their significant role in viral infection and replication [6]. Following viral infection, the host cells express both endogenous and virus-encoded lncRNAs. Some of these lncRNAs exhibit differential expression patterns and impact downstream target gene expression through cis- or trans-regulation; subsequently, they influence viral infection and replication through the modulation of relevant pathways, as well as promoting or antagonizing the expression of antiviral genes [7]. Numerous avian infection-related lncRNAs have been successively identified; for instance, Yanghua He et al. revealed that linc-GALMD1 can coordinate the expression of MDV genes and tumor-related genes to regulate immune responses against Marek’s disease virus (MDV) infection [8], thereby facilitating tumor suppression. Shao Chen et al., on the other hand, found that lnc-ALVE1-AS1 mediated the antiviral response induced by endogenous retroviral elements via the activation of TLR3 signaling in the cytoplasm [9], suggesting a crucial role for lncRNAs in avian viral infections, with many as-yet-undiscovered functions.

Specific pathogen-free (SPF) chicken embryos exhibit a high susceptibility to various pathogenic microorganisms and are extensively employed in avian virus research, particularly for NDV [10]. The existing studies have demonstrated that the immune system of chicken embryos initiates development at an early stage and subsequently generates a discernible immune response; consequently, the utilization of the chicken embryos as in vitro models effectively addresses the limitations encountered when conducting in vivo experiments [11]. Therefore, this study aimed to construct infection models through the infection of SPF chicken embryos with the virulent strain NA-1 and the avirulent strain LaSota at 24 h post-infection (hpi) and 36 hpi. RNA sequencing (RNA-Seq) was performed on infected chicken embryonic visceral tissues to process and analyze transcriptome data, thereby investigating the role of lncRNAs in the pathogenic process of CEVTs when infected with virulent or avirulent NDV strains. To our knowledge, there is a lack of reports regarding host lncRNAs and their biological functions during the NDV virulent infection of CEVTs. Our findings will serve as a valuable resource for comprehending the regulatory functions of lncRNAs in terms of differences related to infectivity and pathogenesis between virulent and avirulent NDV strains. Additionally, this study will contribute towards annotating the chick embryo genome while enhancing our understanding of NDV pathogenesis, ultimately facilitating the development of new vaccines and other control strategies.

## 2. Materials and Methods

### 2.1. Chicken Embryo Challenge and Tissue Collection

The handing and tissue collection procedures for chicken embryos were described in our previous work [12]. In brief, sixty-four 10-day-old chicken embryos were randomly allocated into two groups, one with a single dose of 7 × 10^3^ TCID50 of either virulent strain NA-1 (*n* = 32) and another with an avirulent vaccine strain called LaSota (*n* = 32). Visceral tissues were chosen from chicken embryos using a hemagglutination titer (*n* ≥ 4) in the 24 and 36 hpi groups after NA-1 and LaSota infection for high-throughput sequencing.

### 2.2. RNA Isolation, Construction of cDNA Library and Sequencing

Total RNA extraction from chicken embryo visceral tissues (CEVTs) without any modification was previously described in detail in our study [12]. Thereafter, the qualified RNA samples for library construction underwent tRNA and rRNA removal treatment before cDNA library preparation to meet the specified requirements. The preliminary cDNA library was further processed by performing cDNA end repair and Ploy A tail ligation—an essential component of lncRNA—followed by PCR reaction to obtain a complete cDNA library. This was performed according to the manufacturer’s instructions, without any modification (Illumina Stranded Total RNA Prep with Ribo-Zero Plus kit, Illumina, San Diego, CA, USA). After the purification and quality assessment of the constructed libraries, lncRNA sequencing was conducted on an Illumina NextSeq 2000 Sequencing System (Illumina, San Diego, CA, USA).

### 2.3. Quality Assessment and Splicing Quantification of LncRNA-Seq

The raw data quality values and other relevant information were tallied. This was followed by the visual evaluation of the sequencing data’s quality using Fast QC (version 0.11.2). To ensure accurate information analysis, the raw data underwent processing with Trimmomatic to obtain clean reads. Subsequently, the quality-controlled sequencing sequences were aligned to the reference genome using HISAT2 (version 2.1.0), and statistical alignment was performed using RSeQC (version 2.6.1). Finally, StringTie reference annotation-based transcripts (RABT) were utilized to assemble the genomic mapping results of each sample. Assembly results from multiple samples were then consolidated, discarding transcripts with low levels of expression (FPKM < 1, TPM < 1) during the merging process.

### 2.4. LncRNA Transcript and Expression Analysis

Using the transcriptome assembly results, a series of stringent screening criteria were established based on the structural characteristics of lncRNAs and the functional characteristics of non-coding proteins. We performed (1) exon number screening: low-confidence single-exon transcripts in the transcriptome assembly results were filtered out, and only transcripts with more than two exons were selected. We performed (2) transcript length screening: transcripts shorter than 200 bp were excluded. We performed (3) screening for known transcript annotations: overlapping transcripts with annotated exon regions from the database were identified based on class codes (j, i, u, o, y, x). We performed (4) coding potential screening: the resulting transcripts underwent coding potential analysis using mainstream methods such as CPC2, CNCI, Pfam, and PLEK in order to identify those without coding potential. The intersection of these software’s analysis results was used to generate a predicted lncRNA dataset, which was then statistically analyzed and displayed in terms of their genomic positions.

To quantify NDV infection strength levels based on gene expression, StringTie was used to calculate per million transcript (TPM) values. DESeq2 was employed for further analysis by selecting lncRNAs with q-values ≤ 0.05 and |log2 (fold change)| ≥1, indicating significantly differentially expressed lncRNAs (DELs). Additionally, when comparing between two samples without biological replicates or between groups with biological replicates, at least one sample or group should have an expression quantity of five or more lncRNAs for differences in expression to be considered significant.

### 2.5. Target Gene Prediction and Functional Enrichment Analysis

The function of lncRNAs is associated with their neighboring coding protein genes; therefore, we adopted the default approach of predicting the cis-target genes by considering the coding genes within a 10 kb range upstream and downstream of the lncRNA genes. The names of adjacent genes were compiled into tables and inputted into the DAVID database (https://david.ncifcrf.gov/, accessed on 2 May 2022) to perform Gene Ontology (GO) analysis and Kyoto Encyclopedia of Genes Genomes (KEGG) enrichment analysis. Significantly enriched GO terms and KEGG pathways were determined based on a *p*-value threshold of 0.05. Regarding trans (co-expression) target gene prediction, it follows the basic principle that lncRNAs’ function is linked to their co-expressed protein-coding genes. We utilized the Pearson correlation coefficient method to examine the correlation between lncRNAs and protein-coding genes across samples, retaining only those with an absolute correlation value greater than 0.95 (r > 0.95 or r < −0.95). The aforementioned gene names were summarized in a table and subjected to GO analysis and KEGG enrichment analysis using the DAVID database. Among the names listed, significantly enriched GO terms and KEGG pathways were identified based on a *p*-value threshold of 0.05.

### 2.6. Construction and Analysis of LncRNA–mRNA Interaction Network

We performed significant enrichment screening (*p* ≤ 0.05) to identify target GO terms, and selected lncRNA–mRNA interactions based on the differential expression of mRNAs (|log2(FoldChange)| ≥ 1), combining DELs cis- and trans-target genes. Subsequently, we visualized the constructed lncRNA–mRNA co-expression networks based on our data using Cytoscape (version 3.9.1)

### 2.7. Validation of Differentially Expressed LncRNAs by Quantitative Real-Time PCR (qPCR)

To validate the RNA-Seq results, the ABI StepOne Real-Time PCR system (Applied Biosystems, Foster City, CA, USA) was utilized for qPCR analysis. The Primer-BLAST-designed lncRNAs (http://www.ncbi.nlm.nih.gov/tools/primer-blast/, accessed on 16 November 2022) and gene primers were used in conjunction with the Fast Start Universal SYBR Green Master Kit (Roche, Basel, Switzerland). This kit measured the expression levels of three differentially expressed lncRNAs (DELs) and three differentially expressed genes (DEGs). These were randomly selected from lncRNA–target gene relationship pairs annotated via three rounds of RNA-Seq statistical amplification. Relative gene expression was calculated using the 2^−ΔΔCt^ method.

## 3. Results

### 3.1. RNA Sequencing Output and Characterization of Long Non-Coding RNAs

**Figure 1:**
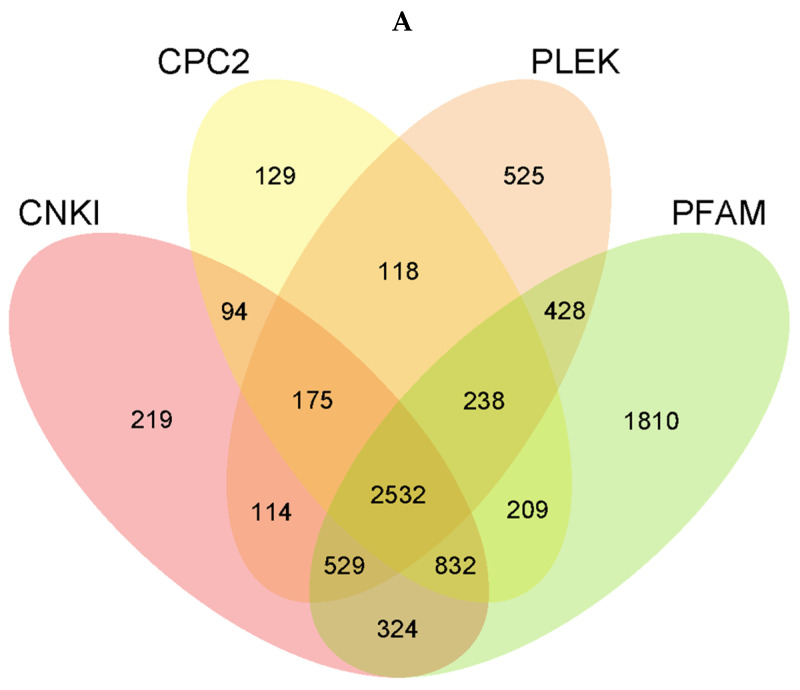

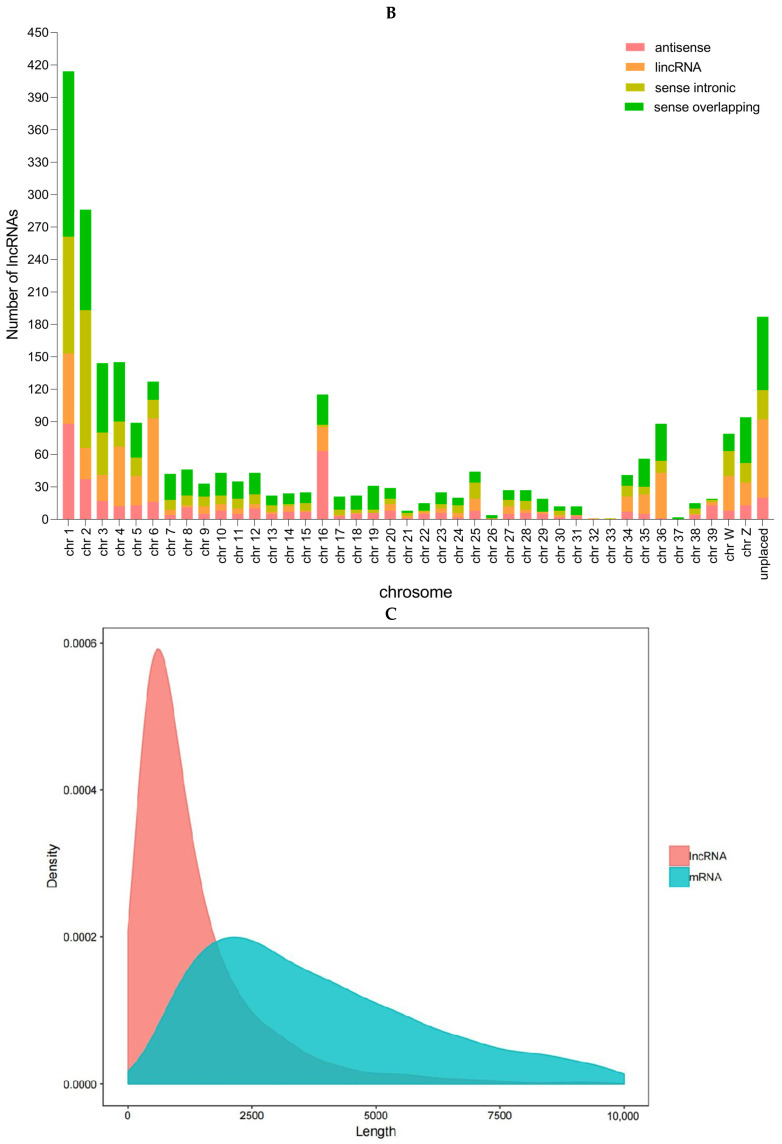

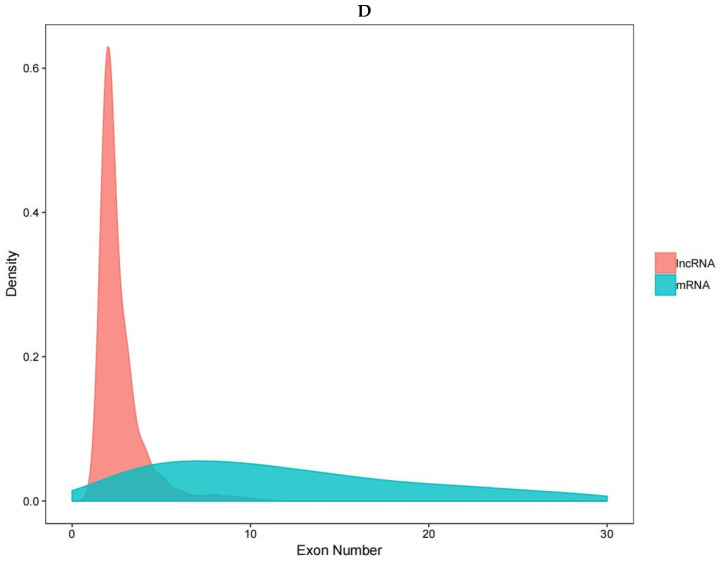
Global lncRNA expression patterns in chicken embryonic visceral tissues after NA-1 and LaSota infection. (**A**) Venn diagram of coding potential prediction. The sum of the numbers in each large circle represents the total number of noncoding transcripts for the software, and the overlapping circles represent the noncoding transcripts shared among the software. (**B**) The statistical map of the distribution of lncRNAs in chromosomes. The horizontal axis is the name of the chromosome, and the vertical axis is the number of lncRNAs located on that chromosome. (**C**) A density plot of exon numbers for comparison between lncRNA and mRNA. The horizontal axis is the number of exons, the vertical axis is the density distribution value, and the different colors represent different types of RNA. (**D**) A comparative density plot of lncRNA and mRNA lengths. The horizontal axis is the length, the vertical axis is the density distribution value, and the different colors represent different types of RNA.

In order to identify the lncRNAs expressed during NDV infection, eight cDNA libraries (N1, N2, N3, N4, L1, L2, L3, and L4) were constructed from the CEVTs infected with NDV NA-1 and LaSota for 24 hpi (N1, N2, L1, L2) and 36 hpi (N3, N4, L3, L4), respectively. Among them, the N1 and N2 libraries represented the NA-1-infected group at 24 hpi, while the L1 and L2 libraries represented the LaSota-infected group at 24 hpi; meanwhile, N3, N4, L3, and L4, respectively, denoted the NA-1- and LaSota-infected groups at 36 hpi. The libraries were sequenced using the Illumina HiSeq™ platform, generating a total of 639,146,390 raw readings across eight cDNA libraries. The raw data were filtered using Trimmomatic to remove sequences containing low-quality sequences with adapters, resulting in 595,283,836 net readings. The percentage of clean reads in each library ranged from 90.96% to 94.79%. We aligned the clean reads to the reference genome, and approximately 57.43–79.71% of the clean reads in all libraries were successfully mapped (Table 1). In addition, strict screening conditions were applied based on transcriptome assembly results and according to the lncRNA structural characteristics and non-coding protein functional characteristics. Four different tools (CPC2, CNCI, Pfam, and PLEK) were used to calculate the coding potential of transcripts for the analysis of the predicted lncRNA dataset. The results revealed a total of 2532 candidate lncRNAs at 1801 sites (Figure 1A). This included antisense lncRNAs, accounting for 444 candidates (17.54%); lincRNAs, accounting for 587 (23.18%); sense_intronic lncRNAs, accounting for 569 (22.47%); and sense-overlapping lncRNAs, accounting for 932 (36.81%). These were distributed across all chromosomes, with the majority found on chromosomes 1 and 2, respectively: 409 (16.2%) and 286 (11.3%) (Figure 1B).

The lncRNAs were further characterized through the comparison of their transcript length and number of exons with those of mRNAs. It was observed that chicken lncRNAs exhibited significantly shorter lengths compared to the mRNAs (Figure 1C). Additionally, the number of exons in lncRNAs was also significantly lower than that in mRNAs (Figure 1D).

### 3.2. Global LncRNA Expression Patterns in Chicken Embryonic Visceral Tissues after Infection with NA-1 and LaSota

**Figure 2:**
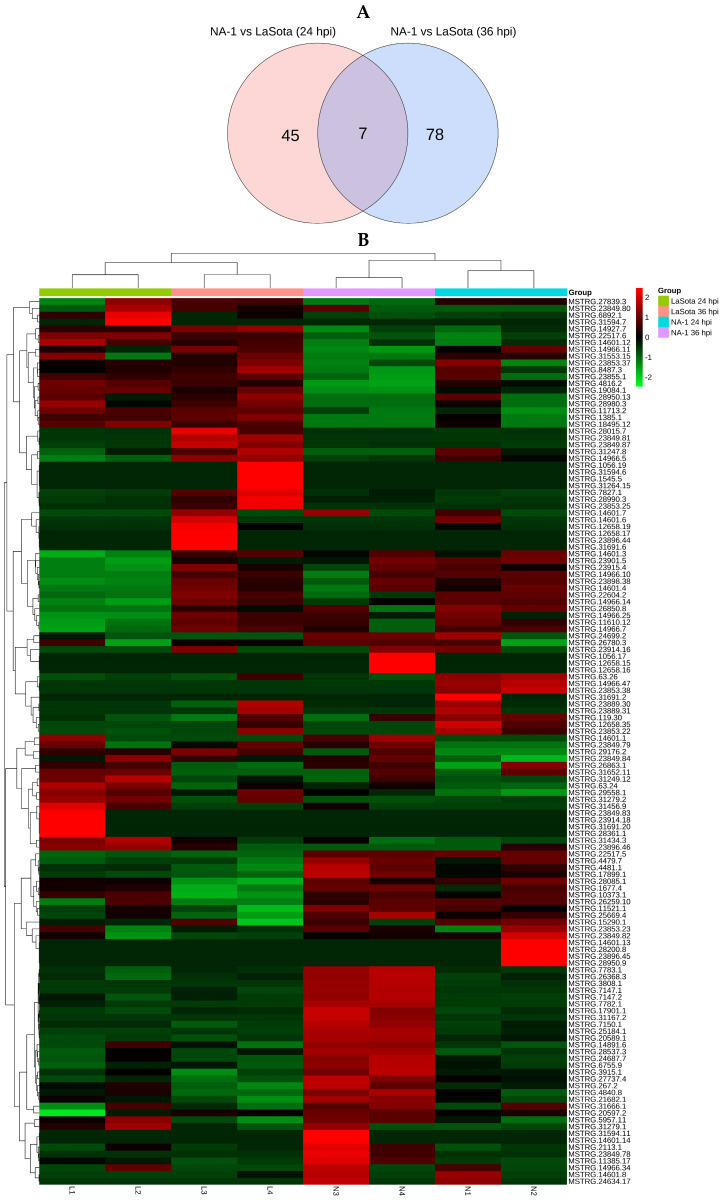

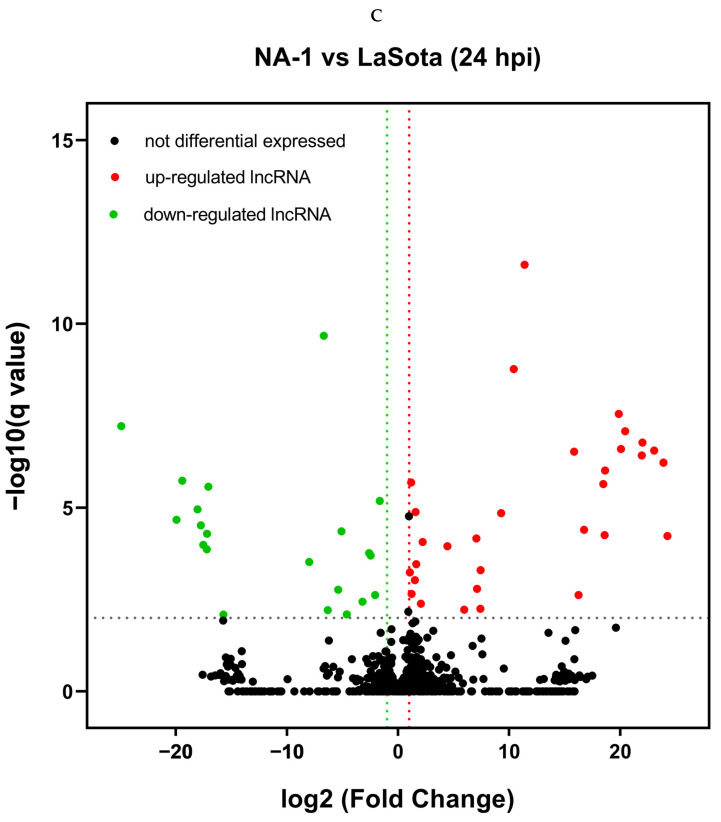

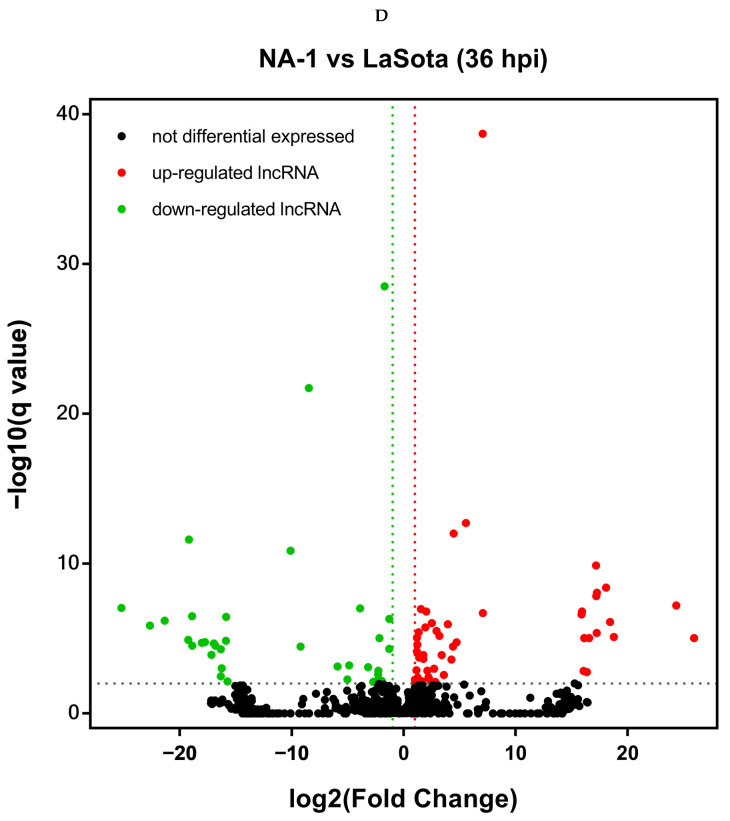
Results from differentially expressed lncRNAs (DELs). (**A**) A Venn diagram of DELs at 24 and 36 hpi. The parts circled with blue and pink represent the DELs at 24 hpi and 36 hpi when NA-1 is compared to LaSota, respectively, and the overlapping parts represent the common DELs in the two comparison groups. (**B**) Heatmap of DELs. DELs were grouped into two clusters according to fold change. (**C**,**D**) A volcano plot of DELs identified in the groups between NA-1 and LaSota at 24 hpi (**C**) and 36 hpi (**D**). The red dots represent up-regulated DELs, and the green dots represent downregulated DELs, respectively.

In order to further investigate the disparities in the infection mechanism of CEVTs caused by virulent and avirulent NDV strains, we employed DESeq2 for sequencing data analysis. To identify significantly different lncRNAs, we established the screening criteria as q value ≤ 0.01 and |log2 (fold change)| ≥ 1. When comparing NA-1 and LaSota strains, there was a total of 52 differentially expressed lncRNAs (including 31 up-regulated and 21 down-regulated) at both 24 hpi and 36 hpi time points (Table S1), while at individual time points, there were 85 DELs (including 50 up-regulated and 35 down-regulated DELs) at 24 hpi (Table S1, Supplementary Materials). The differences in transcript abundance were visually represented using Venn plots as well as volcano plots. Among these DELs, seven lncRNAs exhibited consistent differential expression across both time points, whereas specific expression was observed for forty-five lncRNAs at the earlier time point (24 hpi) and for seventy-eight lncRNAs at the later time point (36 hpi) (Figure 2A). Notably, a significantly higher number of DELs was detected at 36 hpi compared to those detected at 24 hpi. Furthermore, lncRNAs were categorized into two major groups based on the fold change values (Figure 2B).

### 3.3. Interaction Network Construction of Cis- and Trans-Regulated Protein-Coding Genes of lncRNAs

Based on the positional relationship and expression correlation of different lncRNA–mRNA pairs, we predicted the cis- and trans-regulatory genes of lncRNAs to gain a better understanding of their spatial and temporal transcription patterns. This analysis aimed to explore how lncRNAs interact with target genes, participate in regulating the pathogenic differences between strong and weak NDV virulence, and identify the key molecules involved in this process. Therefore, we examined 10 kb upstream and downstream of all identified lncRNAs for protein-coding genes. We discovered that a total of 2532 lncRNAs had 2400 transcripts located near 2881 protein-coding neighbors (<10 kb) (Table S2). Specifically, at 24 hpi, there were 51 DELs, forming 103 cis-regulatory pairs with 64 cis-target genes; meanwhile, at 36 hpi, there were 81 DELs, forming 171 cis-regulatory pairs with 128 cis-target genes.

Furthermore, through co-expression analysis, we predicted the potential targets of lncRNAs in trans-regulatory relationships. We found that, among the analyzed transcripts (2077 lncRNAs) and protein-coding genes (9711 genes), there were significant trans-regulatory interactions (Table S3). When comparing NA-1 vs. LaSota groups, at the time point of 24 hpi, there were 51 DELs, forming 1065 trans-regulatory pairs with 963 trans-target genes (Table S4), whereas at the time point of 36 hpi, we found 78 DELs, forming 2491 trans-regulatory pairs with 2096 trans-target genes (Table S5), which was a significantly higher number than observed at 24 hpi.

### 3.4. GO and KEGG Enrichment Analysis of Genes Regulated by LncRNAs

To further elucidate the response and role of lncRNAs in viral infection at different time points and in response to NDV with varying levels of virulence, we employed Gene Ontology (GO) analysis and KEGG enrichment analysis to functionally annotate the cis-target genes and trans-target genes of differentially expressed lncRNAs in the following comparison groups: Na vs. La (24 hpi-cis); Nb vs. Lb (36 hpi-cis); Na vs. La (24 hpi-trans); and Nb vs. Lb (36 hpi-trans). The cis-target genes and trans-target genes were enriched in multiple biological processes and signaling pathways (Tables S6 and S7). The GO terms and KEGG pathways enriched in trans-target genes were significantly more abundant than those enriched in cis-target genes. Moreover, with increasing infection time, a greater number of genes responded to the infection and participated in various biological processes.

**Figure 3:**
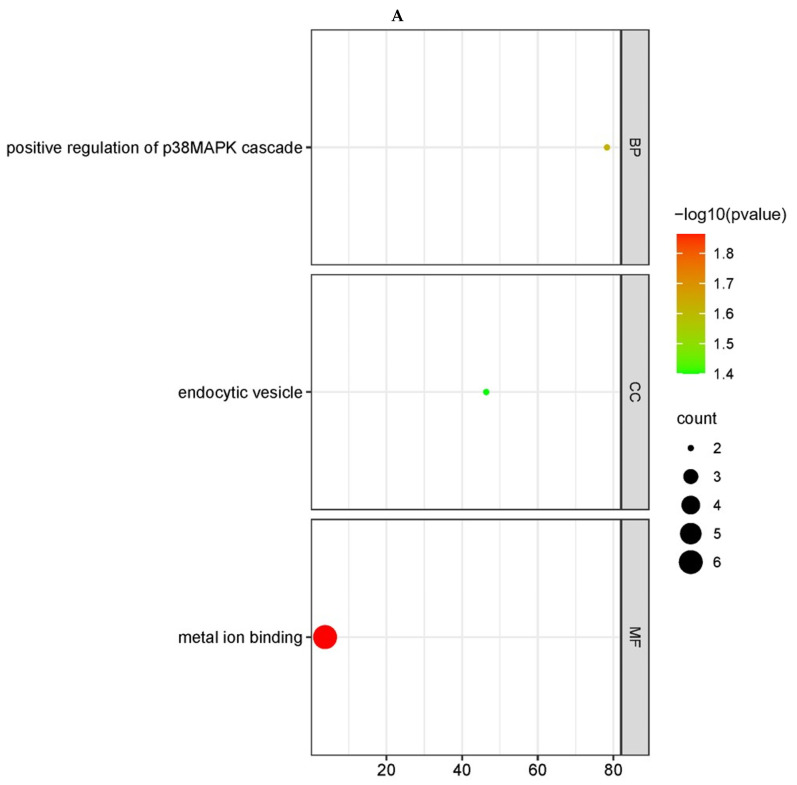

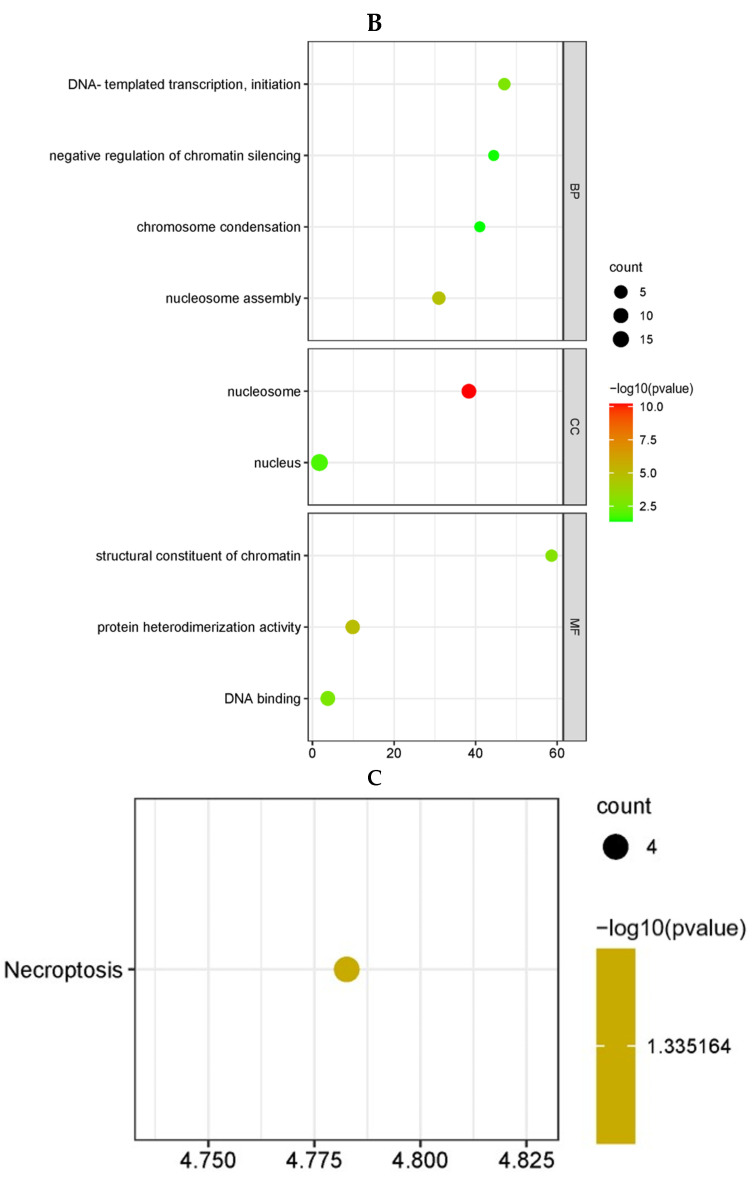
GO and KEGG pathway enrichment analysis of the cis-target genes of DELs in chicken embryonic visceral tissues infected with viruses. (**A**,**B**) GO enrichment analysis of cis-target genes of DELs identified in the groups between NA−1 and LaSota at 24 hpi (**A**) and 36 hpi (**B**). The cis-target genes of DELs, determined according to mean difference (|log2 (fold change)|), are presented and sorted via decreasing mean value (*p* < 0.05). (**C**) KEGG pathway enrichment analysis of cis-target genes of DELs identified in the groups between NA-1 and LaSota at 36 hpi. The dot size indicates the number of cis-target genes of DELs. The redder the color, the smaller the *p*-value (*p* < 0.05).

**Figure 4:**
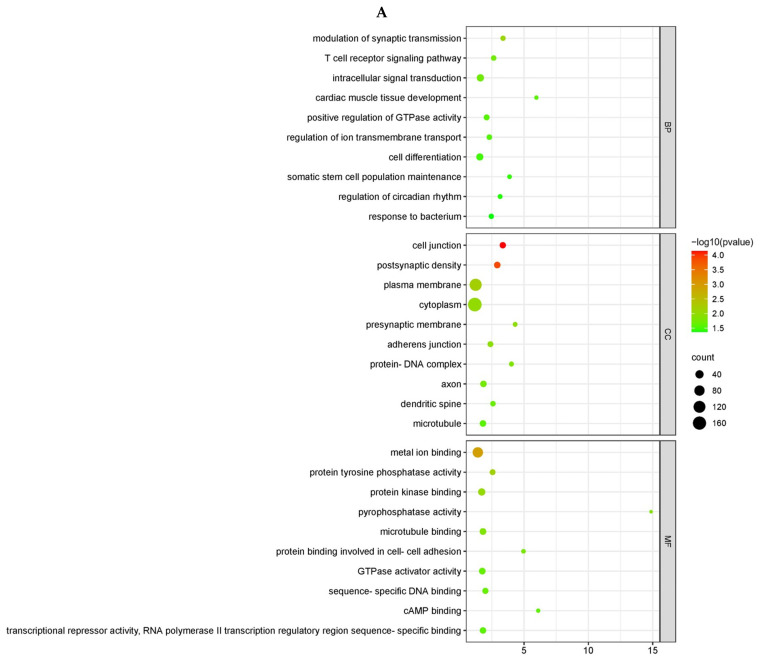

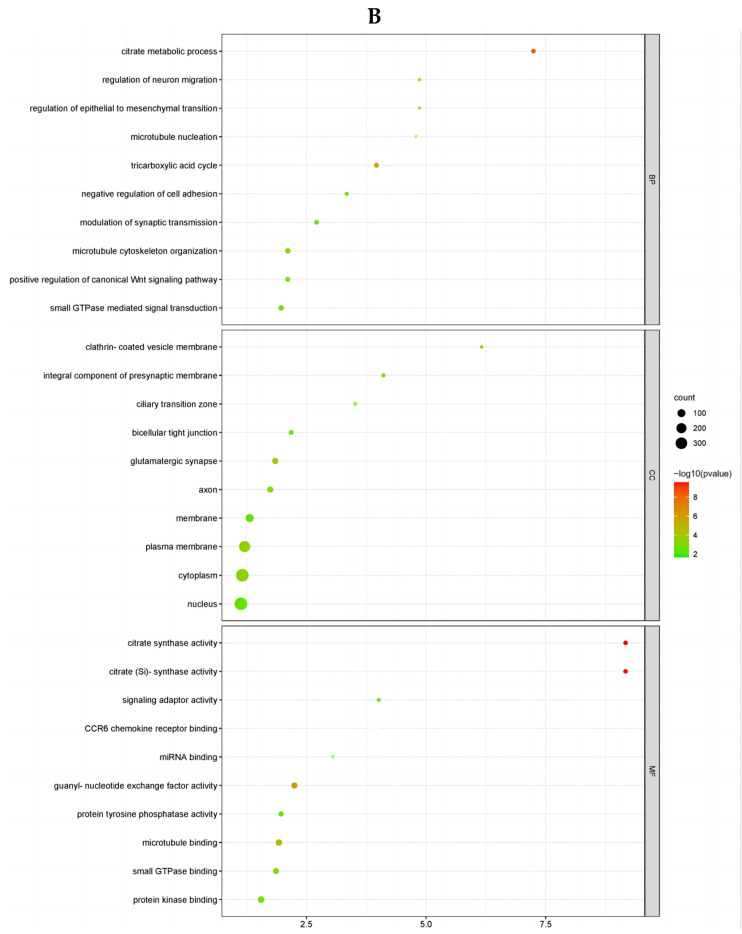

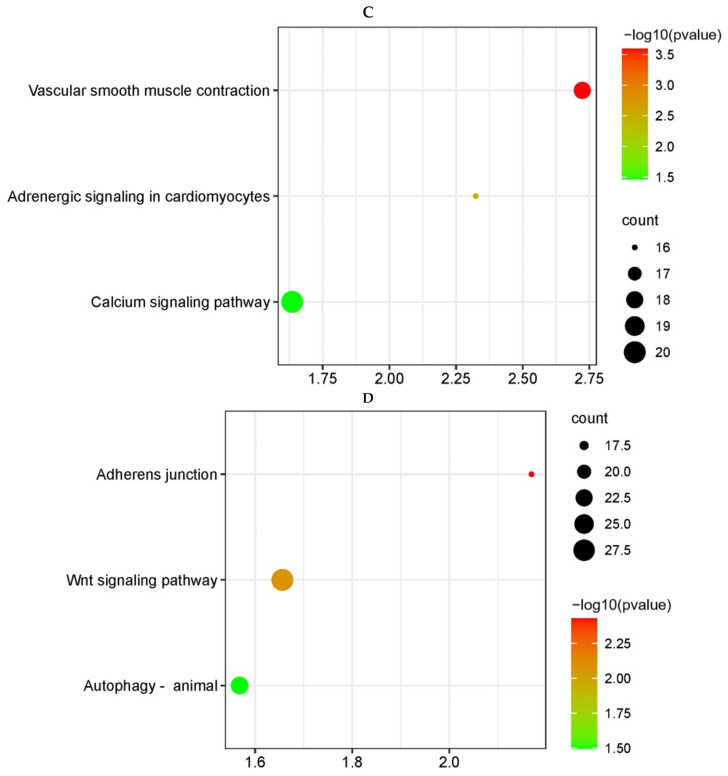
GO and KEGG pathway enrichment analysis of the trans-target genes of DELs in chicken embryonic visceral tissues infected with viruses. (**A**,**B**) GO enrichment analysis of trans-target genes of DELs identified in the groups between NA-1 and LaSota at 24 hpi (**A**) and 36 hpi (**B**). The trans-target genes of DELs, determined according to mean difference (|log2 (fold change)|), are presented and sorted by decreasing mean value (*p* < 0.05). (**C**,**D**) KEGG pathway enrichment analysis of trans-target genes of DELs identified in the groups between NA-1 and LaSota at 24 hpi (**C**) and 36 hpi (**D**). The dot size indicates the number of trans-target genes of DELs. The redder the color, the smaller the *p*-value (*p* < 0.05).

The GO enrichment results revealed that 14 GO terms were significantly enriched among 2 cis-target genes and 111 trans-target genes when NA-1 was infected with LaSota at 24 hpi. When NA-1 was infected at 36 hpi compared to LaSota, a total of 53 GO terms were significantly enriched from 20 cis-target genes and 303 trans-target genes. All four groups exhibited significant enrichment for immune-related items, such as the T-cell receptor signaling pathway, the negative regulation of cytokine production, and the positive regulation of p38 MAPK cascade (Figure 3 and Figure 4). Additionally, except for the Na vs. La (24 hpi-cis) group, the targeted genes in the other two groups were enriched with multiple metabolic-related entries, including the lipid metabolic process, the glycerol-3-phosphate metabolic process, and the tricarboxylic acid cycle. The remaining GO terms were related to different biological processes, such as growth, development, and the nervous system (Figure 3 and Figure 4).

The KEGG enrichment results were relatively limited (Table S8). When NA-1 and LaSota were infected at 24 hpi, a total of 59 trans-target genes showed significant enrichment in five KEGG pathways. In comparison, when NA-1 was infected at 36 hpi compared to LaSota, a total of nine KEGG pathways exhibited significant enrichment for four cis-target genes and one hundred and forty-eight trans-target genes. Notably, no signaling pathways were enriched in the Na vs. La (24 hpi-cis) group. With the exception of the Na vs. La (24 hpi-trans) group (Figure 3C), most of the significantly enriched pathways in the other two groups of target genes were associated with immunity, including the necroptosis pathway, the Wnt signaling pathway, and the autophagy-related pathway. These findings suggest that certain lncRNAs may be implicated in the differences in infection intensity and virulence between NDV strains and contribute to virus–host interactions (Figure 4C,D).

### 3.5. Classification of LncRNA–mRNA Co-Expression and Co-Location Modules Associated with the Infection of NA-1 and LaSota

**Figure 5:**
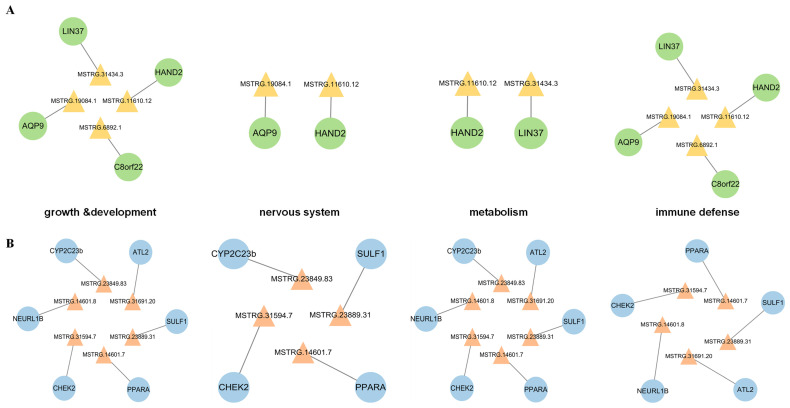

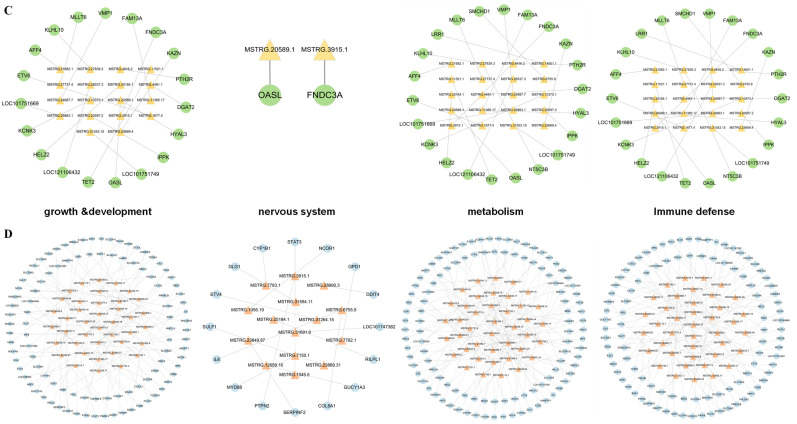
LncRNA–mRNA interaction networks involved in different biological processes. (**A**,**C**) Interactions between DELs and their cis-regulated genes, 24 hpi (**A**) and 36 hpi (**C**). (**B**,**D**) Interactions between DELs and their trans-regulated genes, 24 hpi (**B**) and 36 hpi (**D**). Triangles represent DELs, circles represent mRNAs. There is yellow–green pairing for cis-regulation, and red–blue pairing for trans-regulation.

In order to further investigate the pathogenesis of NDV virulent infection and comprehensively explore the key lncRNAs that interact with DEGs to regulate the host response, we identified DEG involvement in four biological processes—growth and development, nervous system, immunity, and metabolism—based on GO enrichment analysis (Tables S9 and S10). By predicting cis- and trans-target genes of DELs, we constructed regulatory networks by identifying the interactions between DELs and these DEGs (Tables S11 and S12). When comparing the infection of NA-1 with LaSota at 24 hpi, the lncRNA–cis-target gene interaction networks consisted of four DELs and four DEGs. These distinct cis-regulatory relationships were delineated according to different biological processes: four pairs for growth and development, as well as immune processes; two pairs for the nervous system and metabolic processes, respectively (Figure 5A). This network included a total of 26 DELs and 22 DEGs at 36 hpi. It comprised 21 pairs of cis-regulatory relationships for growth and development processes, 2 pairs for nervous system processes, and 26 pairs each for immune and metabolic processes (Figure 5C; Table S11). In a similar way, when comparing the infection of NA-1 with LaSota at 24 hpi, the lncRNA–trans-target gene interaction networks contained six DELs, interacting with six DEGs. These formed diverse trans-regulatory relationships across different biological processes: six pairs for growth, development, and immune processes; and two pairs for the nervous system and metabolic processes, respectively (Figure 5B). At 36 hpi, this network included 41 DELs and 110 DEGs. It formed 103 pairs of trans-regulatory relationships for growth and development processes, 17 pairs for nervous system processes, 101 pairs for immune processes, and 109 pairs for metabolic processes (Figure 5D; Table S12).

### 3.6. Analysis of LncRNAs and Their Target Genes Associated with Multi-Life Process

**Figure 6:**
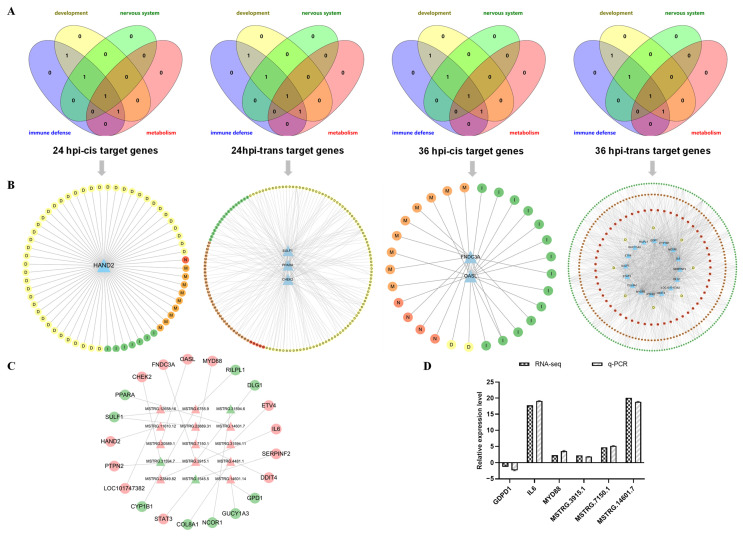
LncRNA–mRNA interaction networks that exist in different biological processes. (**A**) Venn plots of cis- and trans-target genes consistently functioning in multiple biological processes at different time points (the color pink symbolizes immune process, while yellow signifies growth and development. Green represents the nervous system, whereas orange embodies metabolism). (**B**) Functional classification of cis- and trans-target genes enriched to consistently function in multiple biological processes at different time points. Blue triangles represent DEGs, the circles represent biological functions: a yellow circle with the letter D for growth and development: there is a green circle with the letter I for immune response, an orange circle with the letter M for metabolism, and a red circle with the letter N for the nervous system. (**C**) LncRNA–mRNA pairs that continuously function in different biological processes, where triangles represent DELs, circles represent mRNAs, red is up-regulation of expression, and green is down-regulation. (**D**) Validation of RNA-Seq data by qPCR. Expression patterns of selected DEGs and DELs associated with NDV infection of different virulence were detected by qPCR. The y-axis shows expression levels that are normalized to ACTB expression. The x-axis shows the annotations of the selected DEGs and DELs.

The interrelationship and interdependence of numerous biological processes are of significant importance, as certain genes can exert their influence on various biological processes. Hence, based on the constructed lncRNA–target networks (Figure 6B), we identified those DEGs that consistently participated in growth and development, nervous system function, immunity, and metabolism across the four groups. Through a detailed consideration of their GO enrichment results, we selected specific lncRNAs that targeted these DEGs (Table S13). In total, 22 DEGs were identified among the four groups: one in the 24 hpi-cis group and three in the trans group; two in the 36 hpi-cis group; and seventeen in the trans group. These genes exhibited diverse functions within each of the four biological processes (Figure 6A,B). A regulatory network was constructed to illustrate their interactions with corresponding DELs, comprising a total of 22 lncRNA–target gene pairs consisting of 22 DELs and 22 DEGs (Figure 6C).

### 3.7. Validation of RNA-Seq Data Using Quantitative Real-Time PCR

The accuracy of the RNA-Seq data was further confirmed via the random selection of three DELs and three DEGs from the aforementioned 22 lncRNA–target gene pairs for validation through q-PCR analysis. Notably, consistent expression patterns were observed for all selected DELs and DEGs in both RNA-Seq and qPCR analyses, providing strong evidence for the validity of the RNA-Seq data (Figure 6D; Table S14).

## 4. Discussion

### 4.1. NA-1 Infection with NDV Alters Transcript Levels of LncRNAs in Chicken Embryonic Visceral Tissues Compared to LaSota

Virulent strains typically induce severe damage to the various tissues and systems of the body, resulting in a high mortality rate. In contrast, avirulent strains usually only elicit atypical symptoms, or even an asymptomatic infection with a low mortality rate. Therefore, studying the disparities between virulent and avirulent strains of NDV infection is instrumental in elucidating the precise pathogenic mechanisms of NDV and facilitating its prevention and treatment. Furthermore, promoting the advancement of the poultry industry holds immense commercial significance.

Host cells express both their own lncRNAs as well as virus-encoded lncRNAs to modulate viral infection and replication through the regulation of relevant pathways, either enhancing or counteracting the expression of antiviral genes [13,14,15]. Previous studies on NDV infection transcriptomics have predominantly focused on miRNA, mRNA, siRNA, etc., while the knowledge regarding lncRNAs remains limited. However, recent years have witnessed continuous exploration into lncRNAs and their mechanisms of action, revealing their profound importance in viral infection and replication [16]. For instance, Shihao Chen et al. discovered that lncRNA lnc-LTR5B can regulate avian leukemia virus subtype J replication in chicken cells through interaction with BiP; similarly, it was found that lncRNA-LNC_007 exerted an inhibitory effect on NDV replication. The differentially expressed lncRNAs identified herein may also participate in and influence virulence during infection.

Therefore, based on the previous analysis study of protein-coding transcripts, in order to gain a better understanding and elucidate the response of lncRNA levels to host infection with strong and weak NDV strains, as well as explore the potential interaction between lncRNA and mRNA, we further annotated and analyzed the transcriptome sequencing data. In this study, we identified 2532 lncRNAs in the internal organs of chicken embryos infected with NDV at two time points (24 hpi, 36 hpi) using high-throughput sequencing. Compared to protein-coding transcripts, lncRNAs exhibited a significantly shorter length and fewer exons, consistent with their characteristic features. Previous studies have shown that changes in lncRNA expression are closely associated with various biological processes and that their functions are highly correlated with time [17]. Therefore, we screened for differentially expressed lncRNAs at two time points (24 hpi, 36 hpi) in the CEVTs infected with NDV and found 52 DELs at 24 hpi when comparing the NA-1 strain to the LaSota strain; meanwhile, there were 85 DELs at 36 hpi. Interestingly, seven of these lncRNAs showed differential levels of expression at both time points. These differentially expressed lncRNAs may play specific biological roles during the NDV infection process; moreover, their distinct expression patterns at different time points could be related to the dynamic regulation mechanisms underlying NDV infection.

### 4.2. LncRNAs Affect Pathogenicity Differences between Highly Virulent and Avirulent Vaccine Virus by Inducing a Stronger Immune Response in Chicken Embryonic Visceral Tissues

After viral infection, host cells will express both their own and virus-encoded lncRNAs. Among these lncRNAs, certain ones are differentially expressed and regulated by downstream cis- or trans-target genes through immune signaling pathways [18]. This regulation ultimately impacts viral infection and replication, playing a crucial role in the host’s antiviral immune response [19]. Therefore, we selected GO terms and KEGG pathways related to immune responses for further analysis. In both the NA-1 and LaSota groups, lncRNA target genes associated with the host’s innate immune response were induced at 24 hpi and 36 hpi. These genes were significantly enriched via the cis- and trans-regulation of DELs. The enrichment included processes such as the positive regulation of the p38 MAPK cascade and the T-cell receptor signaling pathway, the negative regulation of cytokine production, and the positive regulation of the authorized Wnt signaling pathway along with other GO terms [20,21]. These findings aligned with our previous data from analysis on DEGs, indicating that NDV infection can trigger a robust innate immune response in the host, which contributes to ND pathogenesis [12]. In addition, we discovered that MSTRG.31691.20 was able to trans-regulate multiple target genes, including WNK1, PDE4B, BRAF, FGF1, and PLEKHG5, as well as BRAF again. These genes were found to enrich the T-cell receptor signaling pathway and activate the T-cell receptor signaling pathway, respectively. Different GO terms, such as MAPK activity, indicate that the same lncRNA can regulate host immunity and participate in viral infection via targeting.

Apart from the GO terms and KEGG pathway enrichment genes, several interactions between lncRNAs and target genes were identified as being involved in the host’s innate immune response. Among these DEGs, IL-6 is a key factor in cytokine storm regulation. This is positively regulated by MSTRG.6755.9 after NA-1 infection, leading to its up-regulation. A moderate increase in IL-6 aids in clearing infected cells or damaged tissues, while excessive activation can result in fatal lesions [22,23]. IL8L2 and CX3CL1 are inversely regulated by MSTRG.1545.5 and MSTRG.14601.6, respectively, to regulate inflammatory responses [24]. CD83, CD274, and other leukocyte differentiation antigens are positively regulated by MSTRG.31594.11 and MSTRG.7150.1, respectively, thus promoting antigen recognition and capture, as well as facilitating interactions between immune cells or immune molecules and playing an important role during various stages of immune response activation [25]. In summary, we speculate that lncRNAs may participate in the host’s innate immune response through cis- and trans-actions on related target genes, thus influencing differences in the virulence of NDV.

In addition, programmed cell death also plays a crucial role in the antiviral response through inducing dysfunction or apoptosis of infected cells and neighboring uninfected cells [26]. After their specific induction at 36 hpi, MSTRG.1545.5, MSTRG.14601.6, and other multiple differentially expressed lncRNAs targeted BID [27,28], JAK1 [29], ATG13, ATG14 [30], EIF2S1 [31], and other genes associated with programmed cell death [32]. These lncRNAs are enriched in pathways related to the hepatocyte apoptotic process, necroptosis, and the autophagy–animal pathways. It is speculated that, as the infection progresses, certain lncRNAs become activated in order to regulate their target genes involved in programmed cell-death-related pathways. This dual mechanism inhibits viral replication while promoting virus-induced damage through the inflammatory response triggered by NDV infection. These factors may contribute to the enhanced pathogenicity observed in some virulent NDV strains. Following the viral infection of the host, an appropriate immune response aids in viral clearance; however, excessive activation of antiviral immunity can lead to fatal host damage [23]. Our study suggests that virulent NDV infection induces the expression of specific lncRNAs, within both the host and virus itself, that participate in regulating host immune responses and the induction of antiviral factors, thereby promoting ND pathogenesis through the overactivation of innate immune responses and hijacking the mechanisms underlying programmed cell death.

### 4.3. LncRNAs Affect Pathogenicity Differences between Highly Virulent and Avirulent Vaccine Virus by Altering the Metabolism of Visceral Tissues in the Chick Embryo

Viruses lack their own metabolic networks. After long-term co-evolution, viruses have developed various mechanisms to allow them to interact with the host’s metabolic system, exploiting the host’s metabolic resources for replication by disrupting key metabolic pathways and targeting major regulatory proteins [33]. Additionally, there exists a reciprocal regulation between viruses and innate immunity. Extensive evidence has demonstrated that lncRNAs are intricately involved in cellular metabolic processes, playing a pivotal role in antiviral immunity. For instance, lncRNA-ACOD1 has been observed to be induced by diverse viruses and it also directly binds to the metabolic enzyme GOT2 to enhance its catalytic activity, facilitating influenza virus replication and increasing pathogenicity [34]. However, the utilization of cellular metabolism in different virulent NDVS strains and the question of whether the resulting changes in metabolism are associated with ND pathogenicity and pathological alterations remain poorly investigated.

When comparing NA-1 and LaSota, lncRNA target genes were significantly enriched for multiple GO terms involved in the metabolism of three major substances, including lipid metabolic process, the tricarboxylic acid cycle, carbohydrate metabolism, etc. Among them, IREB2, as the main transcription factor regulating iron metabolism, is involved in the regulation of ferroptosis [35] and is enriched in multiple metabolic processes under the trans-regulation of MSTRG.7150.1. Additionally, CS that is trans-regulated by MSTRG.23849.78 is also enriched in multiple metabolic processes, with its encoded proteins being present in almost all cells capable of oxidative metabolism; they are widely distributed and rich in functions. In addition to GO enrichment and KEGG pathway enrichment gene findings, we observed that multiple DEGs were involved in host metabolic processes. Among them, nine solute carrier superfamily (SLC) genes, including SLC2A14, SLC4A1, and SLC38A2, were up-regulated by MSTRG.28990.3, MSTRG.6755.9, MSTRG.1545.5, and nine other lncRNAs, which regulated the expression of the genes differentially. Solute carrier transporters have a wide range of functions and can regulate lymphocyte signaling through various metabolic pathways while participating in antiviral immunity [36]. Moreover, studies have shown that NDV can promote its replication through up-regulating the expression of SLC1A3 [37].

In summary, it can be speculated that the avirulent LaSota infection only causes a slight metabolic disorder in the host, while virulent NA-1 infection activates certain lncRNAs which act on downstream target genes to manipulate the host’s cell metabolism and utilize its resources to support viral infection. Moreover, the increase in infection time leads to a continuous rise in viral load, which aggravates the viral hijacking of the host’s metabolic resources, resulting in fatal lesions due to an overactivated immune response.

### 4.4. LncRNAs Affect Pathogenicity Differences between Highly Virulent and Avirulent Vaccine Virus by Modulating Organismal Growth and Development, Especially the Nervous System of Visceral Tissues in the Chicken Embryo

The virulent infection of NDV can cause severe damage to chicken embryos and birds during their developmental stage. This leads to significant impairments in the growth and development of various body systems, resulting in deformities and even mortality. A robust antiviral response particularly affects the developing nervous system, which contains non-renewable populations [38]. Furthermore, a virulent strain infection can cause substantial damage to the avian nervous system, with some infected birds exhibiting symptoms of non-suppurative encephalitis. Conversely, weak strain infections may result in no or only mild neurological symptoms.

Our GO and KEGG enrichment analysis revealed that lncRNA target genes related to host growth and development were induced in both the NA-1 and LaSota groups at 24 hpi and 36 hpi. These target genes were significantly enriched through cis- and trans-regulation by DELs. Specifically, GO terms such as cardiac muscle tissue development and cartilage development suggest that lncRNAs are primarily involved in regulating muscle and bone development. Items related to the nervous system mainly pertain to signaling pathways involved in synaptic transmission modulation, such as the semaphorin–plexin signaling pathway, the nerve growth factor signaling pathway, among others.

It is evident that lncRNAs primarily regulate genes involved in synaptic function. These serve as the functional connection between neurons and play a crucial role in information transmission. Abnormalities in synapses can lead to neurological lesions and associated symptoms [39]. Notably, MSTRG.12658.15 regulates nerve growth factor (NGF). This is encoded by the NGF gene, which plays a vital role in neuron survival, differentiation, growth, repair, and regeneration at various stages. Additionally, NGF participates in immune-related signaling pathways, such as the MAPK pathway, NF-κB pathway, and the JNK-p53-Bax apoptosis pathway, to establish a link between the nervous system and the immune system [40]. Furthermore, our regulatory network analysis revealed that MSTRG.6755.9 regulates IL-6, while MSTRG.1545.5 regulates STAT3; STAT3 activates the NF-κB pathway, while IL-6 is targeted by it. Both are pivotal in facilitating the blood–brain barrier permeability required for the transportation of activated immune cells/factors from systemic circulation into the central nervous system/brain parenchyma [41].

Taking into consideration the hemorrhagic lesions observed in hypervirulent avian embryos, particularly in the cerebral region, we hypothesize that, compared to their hypervirulent counterparts, lncRNAs may be up-regulated in hypervirulent avian embryos, modulating embryonic growth and development with a specific focus on neurodevelopment. On one hand, these lncRNAs are implicated in the regulation of synaptic signal transmission, thereby perturbing neural homeostasis. On the other hand, the lncRNA-induced disruption of the blood–brain barrier results in cytokine extravasation into the brain tissue and triggers severe neurological symptoms as well as fatal encephalopathy through an exaggerated immune response.

### 4.5. LncRNAs Persistently Affect Multi-Biological Processes to Induce Pathogenicity Differences between Highly Virulent and Avirulent Vaccine Virus

The biological processes in the body are not isolated but interconnected and interdependent, forming regulatory networks through the coordination and cooperation of numerous proteins, genes, nucleic acids, and other components. Within these networks, certain nodes play pivotal roles that have significant implications across various biological processes and may be closely associated with viral infection. Therefore, we conducted a screening of mRNAs and lncRNAs that consistently participate in four key biological processes: growth and development, nervous system function, immunity, and metabolism. These networks comprise 22 selected mRNAs and 22 lncRNAs. Through this analysis, we isolated 22 pairs of lncRNA–mRNA relationships. Among them, MSTRG.3915.1, MSTRG.7150.1, MSTRG.6755.9, and MSTRG.1545.5 regulate multiple DEGs involved in diverse biological processes. Based on our findings, we propose that viral infection can induce alterations in the expression of specific host lncRNAs, which subsequently regulate downstream target genes involved in diverse functions across different biological processes. This dysregulation ultimately leads to more extensive damage within infected hosts.

## 5. Conclusions

In conclusion, we have elucidated the expression profile of lncRNAs in CEVTs infected with the virulent NDV strain NA-1 and the avirulent strain LaSota through RNA-Seq analysis. We have identified and characterized lncRNAs that may influence NDV, revealing the regulatory relationship between lncRNAs and mRNAs. From an lncRNA perspective, this study has further confirmed that highly virulent NDV induces a robust innate immune response and severe metabolic disorders. Additionally, it affects organism growth and development while targeting the nervous system of infected hosts, leading to life-threatening symptoms in severely affected animals. This study will contribute to understanding the regulatory role of lncRNAs in NDV pathology. In future studies, further investigations into lncRNAs’ functions are needed to explore the precise mechanisms underlying host–virulent NDV interactions at a molecular level.

## Figures and Tables

**Table 1:** Statistical comparison results: the details of eight cDNA libraries.

Category	L1	L2	L3	L4	N1	N2	N3	N4
Raw reads	63,185,830	87,459,796	82,119,456	74,796,940	100,987,550	78,012,660	71,990,202	80,593,956
Clean reads	59,809,132	82,903,540	74,696,552	70,189,696	93,498,134	72,493,762	67,209,392	74,483,628
	94.66%	94.79%	90.96%	93.84%	92.58%	92.93%	93.36%	92.42%
Clean bases	8,226,497,815	11,179,215,599	9,837,276,558	9,394,971,507	12,637,411,374	9,844,025,677	9,098,133,501	9,815,789,727
	8.23 G	11.18 G	9.84 G	9.39 G	12.68 G	9.84 G	9.1 G	9.82 G
Total mapped	46,294,524	66,079,664	44,961,818	44,024,603	60,914,300	48,030,990	44,081,783	42,779,015
	77.40%	79.71%	60.19%	62.72%	65.15%	66.26%	65.59%	57.43%

## Data Availability

The data presented in this study are available on request from the corresponding author due to privacy.

## Supplementary Materials

The following supporting information can be downloaded at: https://www.mdpi.com/article/10.3390/microorganisms12050971/s1, Table S1. Detailed list regarding all DELs in the groups of between NA-1 and LaSota at 24 and 36 hpi. Table S2. Cis-target genes of lncRNAs. Table S3. Trans-target genes of lncRNAs. Table S4. Cis-target genes of all DELs in the groups of between NA-1 and LaSota at 24 and 36 hpi. Table S5. Trans-target genes of all DELs in the groups of between NA-1 and LaSota at 24 and 36 hpi. Table S6. GO enrichment of cis-targets of DELs in the groups of between NA-1 and LaSota at 24 and 36 hpi. Table S7. GO enrichment of trans-targets of DELs in the groups of between NA-1 and LaSota at 24 and 36 hpi. Table S8. KEGG pathway analysis of cis-targets and trans-targets of DELs. Table S9. GO enrichment analysis for all DEGs in the groups of between NA-1 and LaSota at 24 and 36 hpi. Table S10. Classification of DEGs associated with four biological processes. Table S11. lncRNA–mRNA cis interaction network in the groups of between NA-1 and LaSota at 24 and 36 hpi. Table S12. lncRNA–mRNA trans interaction network in the groups of between NA-1 and LaSota at 24 and 36 hpi. Table S13. lncRNA–mRNA interaction network that plays a continuous role in multi-biological processes. Table S14. Primers were used in this work for qPCR validation.
